# IBD barriers across the continents: a continent-specific analysis – Australasia

**DOI:** 10.1177/17562848231197509

**Published:** 2023-09-08

**Authors:** Alexander T. Elford, Rupert W. Leong, Emma P. Halmos, Manal Morgan, Kate Kilpatrick, Peter J. Lewindon, Richard B. Gearry, Britt Christensen

**Affiliations:** Royal Melbourne Hospital, Melbourne University, 300 Grattan Street, Melbourne, VIC 3050, Australia; Concord Repatriation Hospital, Sydney, NSW, Australia Macquarie University, Sydney, NSW, Australia; Alfred Health, Melbourne, VIC, Australia Monash University, Melbourne, VIC, Australia; Queensland Children’s Hospital, Brisbane, QLD, Australia; Christchurch Hospital, Christchurch, Canterbury, New Zealand; Queensland Children’s Hospital, Brisbane, QLD, Australia University of Queensland, Brisbane, QLD, Australia; Christchurch Hospital, Christchurch, Canterbury, New Zealand University of Otago, Christchurch, Canterbury, New Zealand; Royal Melbourne Hospital, Melbourne, VIC, Australia Melbourne University, Melbourne, VIC, Australia

**Keywords:** Australasia, inflammatory bowel disease, multidisciplinary care

## Abstract

Australasia, encompassing Australia, New Zealand, and Papua New Guinea, has some of the highest prevalence’s of inflammatory bowel disease (IBD) in the world. The way IBD medicine is practiced varies between and within these countries. There are numerous shared issues of IBD care between Australia and New Zealand, whereas Papua New Guinea has its’ own unique set of circumstances. This review looks to explore some of the barriers to IBD care across the continent from the perspective of local IBD healthcare professionals. Barriers to IBD care that are explored include access to IBD multidisciplinary teams, provision of nutritional-based therapies, the prevalence and engagement of IBD-associated mental health disorders, access to medicine, access to endoscopy, rural barriers to care, Indigenous IBD care and paediatric issues. We look to highlight areas where improvements to IBD care across Australasia could be made as well as address research needs.

## Introduction

Australasia is a diverse region comprising of Australia, New Zealand and Papua New Guinea.^1^ Across the region, there are variations in ethnicities, socioeconomic status, healthcare access, healthcare literacy and cultural approaches to healthcare. This review looks to explore the various barriers to inflammatory bowel disease (IBD) care across Australasia, from the perspectives of an array of IBD healthcare professionals.

## The Australasian context

Australia is the most populous Australasian country, with an estimated population of 26 million people in 2021, followed by Papua New Guinea 9.9 million people and New Zealand 5.1 million people.^2^ Regarding the human development index, which is comprised of education, standard of living and health indices, Australia and New Zealand are rated very high with Papua New Guinea being rated in the medium range.^3^

The ethnicities of regions are diverse. Australia is a majority Caucasian population with significant immigration from Asia, India, Europe and the Middle East as well as its’ indigenous population reflected by the 2021 census.^4^ New Zealand has four main groups of ancestry: Europeans (70.2%), Māori (16%), Asian (15%) and Pacific (8%).^5^ Papua New Guinea has a majority Melanesian ethnicity with Micronesian and Polynesian minority groups.^5^ The majority of Australians and New Zealanders live in urban areas, whereas the majority of Papua New Guineans live in rural settings.

Australia and New Zealand offer universal healthcare through government health insurance schemes. This provides free hospital cover and provides rebates for general practitioner visits, private practice and subsidies for a variety of prescription medications. Patients can also choose to be treated in private practice, which occurs frequently and is usually associated with an out-of-pocket payment. Papua New Guinea offers universal healthcare, however, is significantly under resourced compared to Australian and New Zealand. Papua New Guinea have an increasing number of private healthcare facilities.

There are significant economic ramifications of IBD given it is frequently diagnosed at an early age, has low mortality, is treated with long-term medications and causes considerable work absenteeism. The economic impact of IBD in Australia was substantial in 2012, with an estimated $100 million (Australian dollar, AUD) spent in hospital care, $361 million (AUD) lost due to productivity losses and $2.7 billion (AUD) lost in other economic costs.^1^ The economic burden from IBD in New Zealand is also substantial, with a burden-of-disease report estimating a loss of $255 million (New Zealand dollar, NZD) in 2017 due to healthcare costs and productivity losses.^6^ The economic impact of IBD in Papua New Guinea is unclear.

## Epidemiology of IBD

### Australia

The inflammatory bowel diseases, Crohn’s disease and ulcerative colitis are chronic diseases of the gastrointestinal tract, affecting the whole age spectrum, leading to significant morbidity and reduction in quality of life. Australasia has followed the world trend of the rising incidence and prevalence of IBD^7,8^ and has one of the highest prevalences of IBD in the world. The first prospective-based population study of IBD in Australia found an incidence rate of 29.6 per 100,000 in the region of Barwon, Victoria, in 2008.^9^ A follow-up study found a crude incidence of 24.2 per 100,000 and a prevalence rate of 344.6 per 100,000 as of June 30, 2011.^10^ A cross-sectional study of general practices across Australia performed between 1 July 2017 and 30 June 2019 found the estimated crude prevalence of IBD was 653 per 100,000 patients (Crohn’s disease 306 per 100,0000 patients and ulcerative colitis 334 per 100,000 patients).^11^ The data were collected using Medicine Insight, a large-scale primary care database and thought to encapsulate 9.7% of the Australian population. Higher socioeconomic status was associated with increased risk of having IBD. Remoteness did not have an observable impact on risk of IBD. There were variations amongst states, with the southern states of Tasmania and South Australia having the highest prevalences. Indigenous Australians had lower rates compared to non-Indigenous Australians (Crohn’s disease Odds ratio (OR): 0.62, ulcerative colitis OR: 0.74).

### New Zealand

There are limited population-based epidemiological studies of IBD in New Zealand, but, like Australia, high incidence rates have been described in localised studies with an incidence rate of 39.5/100,000 for IBD, 26.4/100,000 for Crohn’s disease and 12.6/100,000 for ulcerative colitis in Canterbury when last measured in 2014.^12^ Of note, this study found a 1.6 times greater incidence rate than 10 years prior. Although rates of IBD in Māori and Pasifika are less than other groups, recent data from Rotorua suggest IBD is becoming more common in Māori populations.^13^ Increased urbanisation, hygiene measures and uptake of the Western diet are possible causative factors of this increase. There are no published mortality data from New Zealand.

### Papua New Guinea

There is no published data regarding the incidence and prevalence in Papua New Guinea of IBD, Crohn’s disease or ulcerative colitis. Regarding enteric disease, there is a large burden of enteric infections, with diarrhoeal illness contributing to 15% of <5-year-old deaths historically.^14^ This is largely due to a lack of safe drinking water and sanitation. Among the asymptomatic population, one study found high rates of enteric pathogen carriage among regional and remote communities, in particular shigella and norovirus.^15^ The high burden of enteric infections and the resulting focus of attention may contribute to the lack of published data of IBD.

## Multidisciplinary care services

### Australia

The IBDs are chronic relapsing conditions, frequently requiring long-term medical therapy, including complex drugs with safety concerns, which require monitoring and titrating. IBD can lead to a range of complications, including nutritional, psychological, infectious and problems with other organ systems. Therefore, the needs of the IBD population are best served with multidisciplinary care, which is reflected in the Australian IBD care standards.^16^ Crohn’s & Colitis Australia, a large patient support organisation, identified a lack of multidisciplinary IBD services in their audit of quality IBD care standards.^17^ Public hospitals in urban and regional settings (>50 beds) were invited to participate. Of the 71 hospitals that returned the survey, only one hospital had a full IBD team. Seventeen (24%) hospitals had a partial IBD team, 36 (51%) had an IBD helpline, 26 (38%) had an IBD nurse/s and 15 (22%) had access to a named pharmacist. Less than half of these hospitals had a formal IBD meeting to discuss complex cases. They found paediatric services were more likely to have multidisciplinary teams than adult services. The authors hypothesised these numbers likely overestimated the true figures as numerous hospitals declined to participate, citing a lack of resources. All the partial IBD services were in major cities. These shortages in service provision become clearer when compared to the UK’s IBD audit in 2014. Eighty-six percent of services had an IBD nurse (*versus* 38%), 91% of services had an IBD helpline and 70% of services had a partial IBD team (*versus* 24%).^18^ Benefits of partial IBD services were born out in Australian audit.^17^ Partial IBD services were associated with a 22% reduction in hospital admissions, greater safety monitoring, more patient education and were more likely to use laparoscopic-assisted surgical techniques for surgical intervention (55% *versus* 29%). These results are supported by other studies, which have demonstrated that IBD nurses are associated with reduced healthcare utilisation, improved quality of care and healthcare savings.^19–21^

A subsequent online cross-sectional survey assessing IBD standards in Australia was performed in 2018.^22^ The key difference to this survey was that patients, rather than hospitals, were invited to complete the survey. The majority (74.8%) of the 731 patients who completed the survey were satisfied with their IBD care. Multidisciplinary care was again found to be lacking. The minority of patients had access to IBD nurses (32.4%), dietitians (30.9%), IBD pharmacists (26.1%) and psychologists (12%) as part of their treating team. Only 55.4% of participants confirmed their IBD service had a helpline. One of the suggested consequences of the lack of advice lines were the significant rates of overnight admissions amongst the surveyed population (approximately 25%). More than three-quarters of these were unplanned. It was hypothesised that more readily available advice and access to review may decrease the number of unplanned admissions. The need for improvements in the number of multidisciplinary IBD teams and availability of helplines has been recognised by the Australian Government, as demonstrated by the 2019 IBD national action plan.^23^ The first listed priority is a skilled and multidisciplinary IBD workforce with the aim to implement more IBD nursing and allied health roles. The second listed priority was to support local IBD helplines and to implement a national IBD helpline. In addition to creating and improving helplines, it has been recognised that both general practitioners and patients alike need to be made aware of their existence and purpose. These are good initiatives in response to the clear needs of the Australian IBD population. Creating funding for these healthcare positions and training pathways is the main obstacle to fulfilling these priorities.

### New Zealand

New Zealand published its’ first national audit of IBD care in 2020.^24^ There were three parts to this audit with participation rates 75% for part 1 (IBD nursing survey), 73% for part 2 (IBD service survey) and 67% for part 3 (auditing of individual District Health Board). Similar to Australia, there were multiple staffing issues found. There were insufficient gastroenterologist staffing hours per capita, nursing staff were employed for insufficient hours per capita, often job sharing with other roles such as endoscopy nursing, no service had a complete multidisciplinary team and under two-thirds (62.5%) of departments had formal multidisciplinary IBD meetings. The median duration of nurses in the IBD nurse role was 21 (11–36) months with the authors suggesting this reflected the youth and development of the IBD nursing role in New Zealand. All services that responded to the survey had prompt IBD helplines. Only three services provided formal pathways for GPs to follow up their IBD-related concerns.

### Papua New Guinea

IBD services are lacking in Papua New Guinea. The Australian and New Zealand Gastroenterology International Training Association (ANZGITA) has been developing training partnerships in Papua New Guinea, as ANZGITA have successfully done with various neighbouring Southeast Asian and Pacific countries. They have conducted research into the burden of gastroenterological problems in Papua New Guinea as well an assessment of the local workforce’s ability to manage them.^25^ Initial findings include local doctors reporting high burdens of chronic diarrhoeal illnesses; however, there is no reliable data to confirm this. There is a shortage of gastroenterologist expertise with both Port Moresby and ANGAU (Lae) hospitals lacking formal gastroenterology services or physicians interested in the speciality. As a result, the burden that IBD plays in chronic diarrhoeal illness in Papua New Guinea is unclear. There is also concern regarding the expertise to investigate, diagnose and manage IBD in the region. Help from ANZGITA and other interested organisations will hopefully be of great assistance in improving these issues. In addition to providing training in clinical service, their partnership will also foster opportunities for research in the IBD field.

## Nutritional barriers to IBD care

Historically, diet was largely ignored in the management of IBD but is now recognised as being of great importance for optimal patient care. The past decade has shown emergence of greater consideration of diet as reflected in the first diet-specific IBD guidelines, published in 2017.^26^ This focussed mainly on recognition and management of malnutrition in Crohn’s disease and use of exclusive enteral nutrition (EEN) as induction therapy for children with Crohn’s disease. Since then, dietary therapy in IBD has expanded to include diets used in place of EEN, use of diet for symptom management such as functional gut symptoms and symptomatic strictures, pre-surgical nutrition optimisation, supplementary nutritional products and consideration of disordered eating.^27,28^ As with all evolving models of care, there are many barriers in its application. For dietary therapy in IBD, the main barriers for provision of optimal patient care are access to appropriate nutritional information, expertise and challenges in the application of the dietary therapy. Given the complexities and heterogeneity of IBD, ideally all IBD patients would be seen and advised by a dietitian, well-versed in IBD. IBD guidelines recommend that patients have access to a gastroenterology dietitian as part of their IBD service,^29^ but in Australia, only 31% of adult public IBD services have access to a dietitian,^22^ falling short of the Australian IBD care standards.^16^ As there is variability across different states and regions, many adult IBD patients likely have limited access to dietitian services.

Rates of referral to a dietitian for management of gastrointestinal disease are often low, even when there is access to dietitians, despite the treating physician’s acknowledgement of the importance of dietitian input.^30^ Dietitian access amongst paediatric patients is currently being assessed in Australia but is thought to be worse than adults. In New Zealand, a mixed-methods survey conducted in 2020 found multiple issues amongst the provision of dietitian care for IBD patients.^31^ The majority of gastroenterologists (57%) reported there was a lack of dietitians to meet their patients’ needs. Half of surveyed IBD patients (52%) reported they had seen a dietitian; however, numerous patients commented that dietitians needed better knowledge of IBD. Many of the surveyed dietitians thought their knowledge of the nutritional management of IBD was not current (39%) with more than 50% of dietitians reporting that they see IBD patients infrequently. The national audit noted that dieticians were involved with only 20% of their IBD multidisciplinary meetings.^24^ This paints a picture of a lack of IBD expertise and numbers of dietitians in New Zealand. Given a lack of formal gastroenterology services in numerous major hospitals in Papua New Guinea, specialised dietitian access is assumed to be poor. Compounding the issue of a lack of specialised IBD dietitians in Australasia are competing internet-based diets. These often lack an evidence-base and are fraught with testimonials of benefit from restrictive diets, potentially increasing risk of nutritional deficiencies, reduced food-related quality of life and disordered eating,^32^ in an already at-risk population.^33^

A lack of dietetic services will also impact the application of dietary therapy. Difficulties in application of dietary therapy are not necessarily specific to Australasia. Diets used in IBD management are often restrictive in nature – the most restrictive being EEN. While the efficacy of EEN inducing remission for Crohn’s disease in paediatric patients is well-established, rates of efficacy are lower in adults, likely due to poor tolerability.^34^ Emerging diets aimed for treating disease, based on real food, have varying degrees of restriction and most have not been assessed for tolerability, with some notable exceptions.^35,36^ It seems the greater the restriction, the poorer the adherence, as indicated in a trial comparing the highly restrictive diet Specific Carbohydrate Diet to the Mediterranean Diet, which promotes inclusion of many foods rather than just restriction.^37^

Access to dietitians with expertise in IBD is a barrier to care in Australia and New Zealand. Australia and New Zealand have the expertise; however, greater funding for public dietitian roles is needed to meet this area of need. Given the lack of specialised gastroenterology services in Papua New Guinea, specialised IBD dietitian care may be lower on the priority list of needs.

## Mental health

There is a high prevalence of mental illness amongst IBD patients.^38^ Prevalence of anxiety and depression are both higher, particularly in the context of active disease. Despite this, access to mental health services can be limited.

### Australia

An online cross-sectional survey performed in 2016, confirmed a high mental health burden in Australian IBD patients and demonstrated a lack of referral to mental health specialists.^39^ Amongst the 731 respondents, half of respondents reported distress, 15.2% were currently seeing a mental health practitioner and only 12.2% had access to a mental health practitioner as part of their IBD team. Despite the known high prevalence of mental health disorders in this population, only 16.1% of respondents reported being asked by the IBD specialist or IBD nurse about their mental health. There were numerous negative associations found amongst those with distress, including more frequent hospitalisation, presence of active disease, perianal complications and fistulas. They were more likely to be using opioids or corticosteroids.

The high burden of mental illness amongst IBD patients in Australia has been reproduced in other studies^40,41^ Even when a mental health concern has been identified there is a significant barrier to engagement of mental health services in Australia. One online survey found only 21.3% of IBD patients currently with mental illness were receiving mental health support.^40^ The only predictor found for seeking support was low income (<$60,000). An IBD service in regional Western Australia found many of their patients declined to participate in mental health screening despite the recommendation of their treating clinician.^42^ They found many patients thought screening was ‘not needed’ or that their mental health was ‘self-managed/under control’. Of those who were screened, clinicians were concerned that patients were underreporting distress levels.

Barriers to engagement with mental health services in Australia include a lack of IBD clinicians and nurses exploring mental illness, a lack of willingness of patients to engage in mental health services, a paucity of mental health professionals as part of the multidisciplinary IBD teams as demonstrated by the national surveys^17,22^ and the sometimes prohibitive costs of seeing a psychologist. Most psychological services in Australia are privately based and incur fees. Patients can get access to subsidies through their GP; however, there is often a gap the patient is required to pay. This further highlights the potential benefits of publicly funded mental health practitioners in IBD multidisciplinary teams.

### New Zealand

The prevalence of mental health disorders amongst the New Zealand IBD population is less studied than Australia but seems to be just as substantial.^43^ One surveyed IBD cohort reported 31.2% of participants experienced severe depressive symptoms with 21.8% of the cohort experiencing severe anxiety symptoms. There is a paucity of literature regarding IBD patients in New Zealand accessing mental health services and their attitudes towards this. This is an important research topic moving forward.

## Access to medicines

### Emerging challenge with reimbursement of highly specialised drugs in Australia

The Pharmaceutical Benefits Scheme (PBS) of Australia sets the reimbursement price of highly specialised drugs. Australia has been in a leading position in the uptake of these drugs for many years. With innovation, Australian IBD sufferers should see increasingly effective and safer drugs. The pricing of these drugs, however, might result in difficulties in accessing new complex medications in the future, especially new monoclonal antibodies.

The PBS ensures equal and affordable access to medicines for Australians.^44^ Affordability is partly due to new drugs being priced against the current lowest cost comparator within that class. This is, therefore, often at the price of biosimilars. Ongoing price reductions that occur at regular intervals further decrease the lowest cost comparator pricing. Small molecule generic drugs are simple and cheap to manufacture. Therefore, there is increasing likelihood that future monoclonal antibodies will be priced against generic small molecules. Drug companies may not view the Australian market as commercially viable and may not accept the level of reimbursement, which previously occurred for ustekinumab in ulcerative colitis. This might mean newer drugs are not reimbursed and available only as private scripts and could result in Australian IBD sufferers not having access to innovative new drugs in the future. The first small molecule advanced therapy to lose exclusivity will be tofacitinib. Once a tofacitinib generic drug is available, the new lowest price comparator might result in the reduced entry of new advanced therapies. Tofacitinib generic drug is already available in India.

### New Zealand

New Zealand has a universal healthcare system where all citizens receive publicly funded health and disability services. One aspect of this, pharmaceutical expenditure, is managed by the government agency Te Pātaka Whaioranga, the Pharmaceutical Management Agency of New Zealand (Pharmac).^45^ Pharmac decides what medicines are funded and manages New Zealand’s fixed pharmaceutical budget. Funded medicines cost patients approximately $5 NZD an item (depending on the individual pharmacy; up to a maximum of $100 NZD per year) or for non-funded medicines, the patient must pay ‘out of pocket’. Funded infusions are often administered free of charge.

Historically, access to IBD treatments in New Zealand has lagged behind other lower Organisation for Economic Co-operation and Development countries with New Zealand having fewer available funded treatment options.^46^ With intense lobbying ustekinumab and vedolizumab were funded for moderate-to-severe IBD in New Zealand on 1 February 2023 although several oral medications are still not available in New Zealand. This funding change is expected to significantly improve care for many IBD patients in New Zealand however a lack of Pharmac transparency, funding delays and the requirement for ongoing IBD advocacy has meant some IBD patients have received suboptimal IBD care. As novel medications become available in the future, Pharmac funding decisions are expected to remain an ongoing issue for IBD patients’ accessing newer therapies in a timely manner.

### Papua New Guinea

Papua New Guinea has historically had access issues to essential medicines.^47^ This suggests access to specialised biologics and small molecules of IBD would be challenging.

## Endoscopy access

### Australia

Endoscopy plays a key role in both the diagnosis and surveillance of IBD. Most hospitals use a three-tiered triaging system for colonoscopy indications, which are supported by government guidelines in some states.^48–50^ Category 1 usually reflects a concern of cancer diagnosis and requests for dysplasia surveillance procedures or assessment of mucosal healing usually fall under category 2. Category 1, being the most urgent, are ideally meant to be conducted within 30 days of the request. National data from bowel cancer screening suggests category 1 patients often wait longer than 30 days, with a median of 52 days from positive screen to colonoscopy as of 2017.^51^ Given the importance of category 1 procedures, they are often prioritised, usually resulting in comparatively worse waiting times for category 2 and 3 procedures, in many centres across Australia. This problem has escalated since the COVID-19 pandemic, resulting in significant waiting times for most IBD-related endoscopy indications. The problem of disease assessment is partially mitigated at some centres by the growing use and expertise of gastrointestinal ultrasound, which can be useful for evaluating disease activity and monitoring.

The other endoscopy-related issue encountered in Australia has been knowledge deficits and adherence to dysplasia surveillance guidelines for IBD patients.^52–54^ Multiple studies have demonstrated low uptake of advanced screening techniques, in particular dye-chromoendoscopy, which is the preferred screening method of choice as per the Australian guidelines.^55^ Awareness/familiarity with dysplasia guidelines has been lower than expected in some studies. Knowledge of guidelines and use of advanced screening techniques have been shown to be more frequent with gastroenterologists and IBDologists.^54,55^ We note that this is not an isolated problem to Australia with numerous other countries experiencing similar issues.^56–60^ Performance of surveillance colonoscopy in IBD patients is not a training requirement for colonoscopy accreditation in Australia, which may explain some of the above results. We advocate that dysplasia surveillance be arranged and performed by endoscopists with training and interest in this area. The glaring barrier to this will be in regional and remote settings, where many colonoscopies must be performed; however, this expertise will often be lacking.

### New Zealand

There are similar issues in New Zealand for endoscopy access for IBD patients. IBD endoscopy is performed by both gastroenterologists and general surgeons in the New Zealand. New Zealand has national guidelines which detail the expectations for time focused endoscopy targets.^61^ There is a preference for endoscopists with a special interest and or experience in managing IBD to perform these procedures; however, for geographical and scheduling reasons, this is not always possible. COVID-19 has also had an impact on the delivery of endoscopy, and it is expected that many New Zealand IBD patients are overdue for surveillance endoscopy. Despite these challenges, a recent survey of New Zealand endoscopists regarding their approach to IBD dysplasia practice, suggested good adherence to guidelines regarding screening intervals, risk stratification and techniques.^62^

## Rural barriers to care

### Australia

Australia has challenges to delivering equitable healthcare due to its large land mass. The Australian Institute of Health and Welfare reported that the majority of Australians live in major cities (72%), with almost 10% of Australians living in outer regional, remote or very remote areas in 2019.^63^ Access to medical specialists for people in remote areas is challenging, with 58% of participants reporting they did not have access to a medical specialist. Access to multidisciplinary IBD care is challenging and as mentioned above, is a particular problem outside urban areas with no partial IBD units identified in the IBD national audit.^16^ We believe the fulfilment of the IBD national action plan for multidisciplinary teams and the availability of helplines will be of particular benefit to the regional and remote population.^23^ In addition to these priorities, the national action plan aims to foster the ability for rural healthcare professionals (general practitioners, allied health staff and general physicians) to seek advice from more specialised colleagues. The Australian Government would also like to conduct further research and auditing into the prevalence and quality of IBD care in rural Australia, as part of the strategic plan.

Another strategy for improving care is to broaden the reach of existing IBD teams. Telemedicine and the various digital platforms of providing remote healthcare have been effective in delivering healthcare for a variety of medical conditions, including IBD.^64^ For many regional and remote patients, attendance in-person medical appointments require long travel times, significant finances and access to a vehicle. Public transportation is not always available for these appointments, and some areas require transport by boat or by plane. The problems are compounded if the patient is complex and requires specialised investigations such as magnetic resonance imaging and colonoscopy. Ankersen *et al.*^64^ have written a review article citing how Denmark’s use of electronic health (E-Health) would have various advantages in the Australian IBD context. Many patients are well; therefore, a telemedicine appointment is satisfactory for purposes of review, ordering pathology and prescribing medications. More than half of IBD patients in Australia are treated in private practice, where there is often the technology to support telehealth and receive Medicare reimbursements for telehealth consultations. There are widely accessible patient support groups in Australia, for example, Crohn’s and Colitis Australia, who educate and support patients through this model of care. Barriers to providing this care in the Australian context include insufficient digital infrastructure in certain areas, privacy of cloud-based data and the legislated limits on the proportion of telehealth consultations a practitioner can bill the government funding agency, Medicare.

The benefits of telemedicine in IBD have been demonstrated in Australia cohort.^65^ A survey amongst 139 IBD patients who transitioned to a telehealth model of care during the 2020 pandemic found the overwhelming majority (88.1%) of patients were ‘satisfied’ or ‘very satisfied’ with the care they received. Overwhelming satisfaction was also demonstrated with clinic appointment length of time and the input the patients felt they had in their care. The impact on work absenteeism was remarkable, with 60.4% of patients reporting they would ordinarily need to miss work with face-to-face appointments *versus* 20.9% needing to take off time for their telehealth appointments. There were similar rates of non-attendance between the telemedicine *versus* pre telemedicine eras (10.4% *versus* 11.4%). This suggests that telemedicine can work well in an Australian IBD context; however, it is important to note this was conducted in a major metropolitan centre with a comprehensive IBD unit, so further research is needed to evaluate the efficacy in a regional and remote context.

Access to therapeutic monitoring is another challenge for regional and remote patients. In the era of the treat-to-target approach, requests for faecal calprotectin and therapeutic drug levels are ordered frequently. The benefit of these tests is they can be collected at local pathology centres, which are often found in regional areas. Faecal calprotectin has been particularly useful for disease assessment in regional areas of Australia, where access to specialist endoscopy or magnetic resonance imaging may be limited. The problems are these tests are not covered by the Medicare benefits schedule (except for some diagnostic purposes) and can be expensive, particularly when performed numerous times per year. Many large public teaching hospitals in Australia cover the fees for these tests; however, regional and rural patients will usually have to pay an out-of-pocket fee at their local pathology centre.

### New Zealand

Another barrier to IBD care is geographically marginalised groups living in rural and remote areas of New Zealand with these patients often experiencing inequalities in health.^66,67^ In New Zealand, 26% of the population live in rural or small urban areas with a higher proportion being Māori.^68^ This has implications for IBD patients living in remote communities, experiencing difficulties accessing infusion centres for administration of medications as well as access to general practice and specialised gastroenterology care. The availability of subcutaneous self-administered medications helps to mitigate this; however, many patients still require regular intravenous infusions, and most need ongoing specialist input.

Currently, there are four regions in New Zealand that do not have a local gastroenterologist, including Tairāwhiti, Wairarapa, Whanganui and the West Coast.^69^ Patients living in these areas receive IBD care through their general practitioner or must travel significant distances to receive specialist care. For example, patients living in Greymouth, must travel 3 h to Christchurch to attend a specialist gastroenterology appointment. COVID-19 has led to the development of telehealth which improves the provision of health services. Telehealth will be useful at overcoming rural healthcare barriers as well as reducing health disparities for IBD patients.^70^ Similarly, the establishment of Te Whatu Ora – Health NZ in 2022 and the abolishment of District Health Boards may also help address rural barriers to IBD care, with the organisations’ main purpose being equitable and accessible healthcare.

## Equity/Indigenous barriers

### Australia

Whilst Australia has one of the highest prevalences of IBD in the world, the Indigenous population (approximately 5% of total population) has a low prevalence.^71^ The Northern Territory, which has an approximately one-third Indigenous population, has a prevalence of 186/100,000 for non-indigenous *versus* 5/100,000 in the Indigenous populations, respectively.^72^ A measured eight times lower risk rate among the Indigenous population has also been found in a paediatric cohort.^73^

Factors proposed to contribute to this include the hygiene hypothesis, genetics, gut-microbiome composition and the immune response to helminthic infections. Given the low prevalence of IBD, targeting IBD care specifically amongst the Indigenous Australian population did not feature as a strategic goal in Australia’s most recent IBD national action plan.^23^

### New Zealand

Māori have lower rates of IBD compared to non-Māori although incidence rates appear to be increasing. The Canterbury IBD study published in 2006 reported Māori comprise 1% of IBD patients, with more recent population data from Lakes District Health Board reporting 7.6% of IBD patients as Māori.^74^ The rates of Māori with IBD at Lakes District Health Board had an eightfold increase from 2011–2015 to 2016–2020.

Māori health status is significantly lower compared to non-Māori across all domains with health inequalities occurring throughout the healthcare system including reduced accessibility to healthcare services, general practice and lower rates of colonoscopy.^75,76^ The establishment of Te Aka Whai Ora – the Māori Health Authority will continue to address health equity for Māori, driving change to respond to Māori health needs, as will Pharmac who prioritises equity for Māori in funding decisions.

## Paediatric issues

Up to 25% of patients with IBD are diagnosed in childhood.^77^ The incidence and prevalence of paediatric inflammatory bowel disease is increasing worldwide, including Australia and New Zealand.

### Barriers to diagnosis

Prompt diagnosis is impacted by a variety of factors. The gold standard for diagnosis is endoscopy and biopsy with evidence of histological changes. Access to endoscopy is limited by the availability of hospitals with appropriate staffing and procedural equipment. Other diagnostic testing such as magnetic resonance enterography and capsule endoscopy require general anaesthesia to be undertaken in younger paediatric patients, particularly those less than 7 years of age. They also require paediatric specialist availability for interpretation and reporting. These facilities are predominantly available in tertiary centres, which are mostly in capital cities. Given Australia is a vast country, patient access to tertiary paediatric centres is challenging. Although the area of New Zealand is not as vast as Australia, the country is separated over two islands. Each island has a paediatric gastroenterology service. Hence, limited numbers of diagnostic and treating facilities increases wait times for timely outpatient review and endoscopy. Delays and barriers to services impact timely commencement and review of management and therefore disease severity, outcomes and morbidity.

### Barriers to optimal management

#### Access to biologics/novel therapies

Early commencement of appropriate medical management is essential for achieving remission and decreasing long-term complications. Research assessing the safety and efficacy of medications as well as dosing and long-term outcomes is limited in the paediatric population.^78^ This restricts management as there are guarded levels of confidence regarding use and safety profile of many medications, particularly novel ones. Access to the most effective medication is further restricted by government drug subsidy regulations, in particular the requirement for patients to receive and fail immunosuppressive medication, namely thiopurines or methotrexate for a minimum of 3 months prior to qualifying for biological therapy in both ulcerative colitis and Crohn’s disease. Given the more severe and extensive luminal disease that is often observed in the paediatric population, this creates an access barrier to some of the most efficacious medical management which contributes significantly to morbidity. There are often limitations to access newer drugs because of a lack of targeted paediatric pharmacokinetic, pharmacodynamic and safety data. This has been demonstrated by the challenges of accessing vedolizumab. Vedolizumab monotherapy has an excellent therapeutic and safety profile. However, vedolizumab it is not available *via* government subsidy in Australia and can only be accessed on a compassionate basis through the drug company. Company policy limits access to those over 6 years of age or 30 kg. Other agents, such as ustekinumab and tofacitinib are readily available and widely used in the adult population; however, paediatric patients can only access these medications on a compassionate basis, and even with shared funding in the case of ustekinumab, the costs are considerable for the individual gastroenterology departments.

### MDT management

A multidisciplinary approach is crucial to optimal management in the paediatric IBD population and is discussed in detail in the Australian IBD healthcare standards.^16^ Many patients are malnourished and have nutritional deficiencies during this key developmental time. EEN is used more frequently amongst the paediatric population so dietetic support for education, institution and monitoring of patients undertaking EEN for treatment of Crohn’s disease is essential. Co-existing mental health issues are also prevalent within the paediatric population and impact on their quality of life and compliance with treatment.^79^ Graffigna *et al.*^80^ highlighted the importance of early identification and management of mental health issues in patients to ensure optimal patient engagement. Adequate medication education and training is paramount to increase compliance and limit side effects. As such, a skilled pharmacist is essential to the functioning of the team. Most of these services are limited in their availability to the paediatric population, even within a tertiary centre or capital city. When comparing to the adult population, the IBD national audit reported that all surveyed paediatric centres had an IBD helpline and were more likely to have a stoma nurse, IBD nurse, dietician and named pharmacist than the adult centres.^17^

### Transition

**Figure 1.**
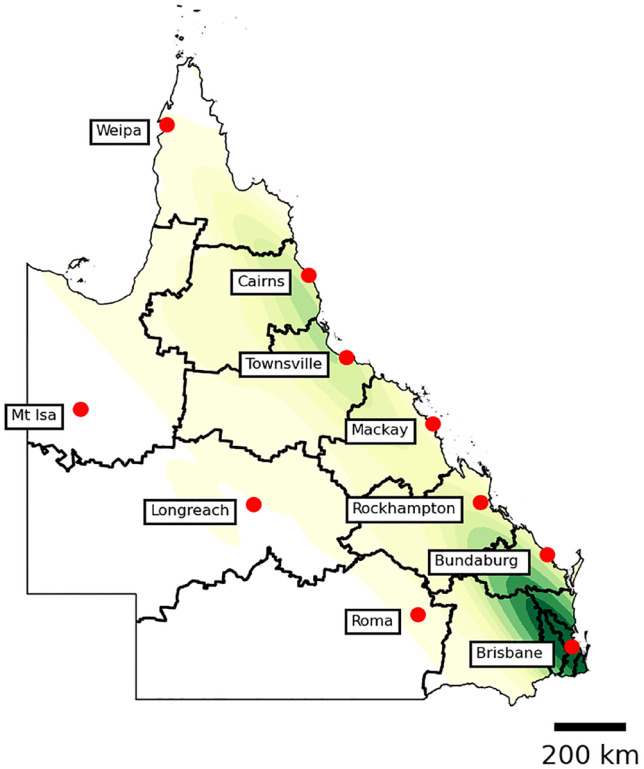
Heat map of referrals from Queensland Children’s Hospital in Brisbane to adult IBD services across the state.

There are several steps that must occur over several years to successfully transition a paediatric patient to adult IBD services. This should begin at age 12–14 years according to Crohn’s and Colitis Australia and involves commencing discussions with the young adult and their guardians around the process.^81^ Optimal transition occurs with responsibility for individual care being transferred from parents/guardian to the patient as well as from paediatric to adolescent centre. Despite extensive evidence of the importance of dedicated transition clinics and processes for successful transition, access is limited as demonstrated by the national audit (16/66 hospitals had transition services),^17^ and many patients are simply transferred directly to adult services. As there are differences in healthcare models and delivery of paediatric and adult IBD care, the patient needs to be adequately prepared for transition to ensure ongoing engagement and to limit the impact of their disease and management on quality of life at this time.^82^ The disease phenotype in paediatric patients characteristically becomes more complicated as patients reach adolescence. Hence, transition from paediatric to adult care requires a collaborative approach which minimises gaps in care. Additionally, plans post-secondary education, such as travel, tertiary studies and new employment of this patient group during the period of transition adds an extra layer of complexity to successful transition process and the determination of where, in fact, the patient will transition to, not always the same locality as their childhood. Lastly, major logistical challenges occur in referring paediatric patients from a small number of tertiary, capital city-based paediatric centres to a large number of public services and private practitioners scattered over a large land mass, each with different triage processes and age at acceptance of referrals. An example of this is barrier is the state of Queensland. Queensland’s Children’s Hospital is the state’s major specialist paediatric hospital, located in the state’s capital, Brisbane. The IBD service serves the entirety of Queensland and has to transition patients to a multitude of adult services across the large state as demonstrated by Figure 1. This alone makes standardisation of the transition process almost impossible. Telehealth offers a means to bridge the challenges provided by distance.

## Conclusion

Australia and New Zealand do IBD care well in many ways; however, there are numerous outstanding issues. Access to specialised IBD multidisciplinary units, including the full complement of allied health professionals, is an important barrier to care. Dietary assessment and therapy are becoming increasingly important in the IBD treatment paradigm; however, there is a significant lack of dietitian funding, which hinders the delivery of specialised nutritional care in the Australasian context. There are significant rates of mental health disorders within the Australasian IBD context, which is compounded by a lack of mental health professionals within IBD teams. These IBD multidisciplinary issues are exacerbated in regional and rural settings; however, telemedicine provides a promising avenue to improve this area. Access to specialised IBD care may be more difficult amongst the paediatric population where specialised IBD care may be limited to select tertiary centres. Additionally, well-supported transition models of care are lacking. Whilst there is access to biologicals in Australia and New Zealand, there are numerous anticipated barriers to access newer biologicals and small molecules given the respective governments funding schemes. Surveillance procedures for endoscopy have been substantially delayed since the COVID-19 pandemic, and access to specialised endoscopists remains an ongoing problem. Papua New Guinea has a shortage of gastroenterologists and a paucity of data regarding IBD.

## Declarations

**Ethics approval and consent to participate:** Not applicable.

**Consent for publication:** Not applicable.

**Availability of data and materials:** Not applicable.
